# Adolescent Female Victims of Sexual Violence: Analysis of Loss of Follow-up after Emergency Care and Outpatient Follow-up

**DOI:** 10.1055/s-0043-1772594

**Published:** 2023-11-29

**Authors:** Alejandra Suyapa Becerra Torres, Otávio Prado Alabarse, Ândria Cléia Alves, Ana Luiza Teixeira, Renata Cruz Soares de Azevedo, Arlete Fernandes

**Affiliations:** 1 Universidade Estadual de Campinas, Departamento de Tocoginecologia, Divisão de Ginecologia, Campinas, SP, Brazil

**Keywords:** loss to follow-up, adolescence, rape, sexual violence, retrospective study, perda de seguimento, adolescência, estupro, violência sexual, estudo retrospective

## Abstract

**Objective** To assess the loss to follow-up after emergency care and during 6-months of outpatient follow-up, and the associated variables, among adolescent sexual violence survivors.

**Methods** This is a retrospective study with review of the medical records of 521 females, aged 10 to 18 years, who received emergency care in a referral service in São Paulo, Brazil. The variables were sociodemographic; personal history; characteristics of abuse, disclosure, and reactions triggered after abuse (physical and mental disorders as well as social changes), psychotropic prescription needs, and moment of abandonment: after emergency care and before completing 6 months of outpatient follow-up. To compare groups of patients lost to follow-up at each time point, we used the Chi-square and Fisher exact tests followed by multiple logistic regression with stepwise criterion for selection of associated variables. We calculated the odds ratio with confidence interval (OR, CI 95%). The level of significance adopted was 5%.

**Results** A total of 249/521 (47.7%) adolescents discontinued follow-up, 184 (35.3%) after emergency care and 65 (12.4%) before completing outpatient follow-up. The variables of living with a partner (OR = 5.94 [CI 95%; 2.49–14.20]); not having a religion (OR = 2.38 [CI 95%;1.29–4.38)]), having a Catholic religion [OR =  2.11 (CI 95%; 1.17–3.78)]; and not disclosing the abuse [OR = 2.07 (CI 95%; 1.25–3.44)] were associated with loss to follow-up after emergency care. Not needing mental disorder care (OR = 2.72 [CI 95%; 1.36–5.46]) or social support (OR = 2.33 [CI 95%; 1.09–4.99]) were directly associated with loss to outpatient follow-up.

**Conclusion** Measures to improve adherence to follow-up should be aimed at adolescents who live with a partner and those who do not tell anyone about the violence.

## Resumo

**Objetivos** Avaliar a perda de seguimento de adolescentes vítimas de violência sexual após o atendimento de emergência, durante o seguimento ambulatorial e as variáveis associadas.

**Métodos** Estudo retrospectivo com a revisão de prontuários de 521 mulheres de 10 a 18 anos, que buscaram atendimento de emergência em um serviço de referência em São Paulo, Brasil. As variáveis foram sociodemográficas; antecedentes pessoais; características do abuso, atitude de revelação e reações desencadeadas após o abuso (distúrbios físicos, mentais e mudanças sociais), necessidades de prescrição de psicotrópicos e momento do abandono: após atendimento de emergência e antes de completar 6 meses de seguimento ambulatorial. Para comparar os grupos de perda de seguimento em cada momento, foram utilizados os testes do qui-quadrado e exato de Fisher, seguidos de regressão logística múltipla com critério *stepwise* para seleção das variáveis associadas. Calculamos a razão de probabilidade com intervalo de confiança (RP, IC 95%). O nível de significância adotado foi de 5%.

**Resultados** Um total de 249 (47,7%) das adolescentes descontinuaram o acompanhamento, 184 (35.3%) após o atendimento de emergência e 65 (12.4%) antes de completar o seguimento ambulatorial. As variáveis de viver com companheiro [RP = 5,94 (IC 95%; 2,49–14,20]; não ter religião [RP = 2,38 (IC 95%;1,29–4,38)], ter religião católica [RP =  2,11 (IC 95%; 1,17–3,78)] e não revelar o abuso [RP = 2,07 (IC 95%; 1,25–3,44)] foram associadas à perda de seguimento após o atendimento de emergência. Não necessitar de cuidados de saúde mental (RP = 2,72 [IC 95%; 1,36–5,46]) ou apoio social (RP = 2,33 [IC 95%; 1,09–4,99]) foram as variáveis associadas à perda do seguimento ambulatorial.

**Conclusão** Medidas para melhorar a adesão ao seguimento devem ser direcionadas às adolescentes que vivem com parceiro e às que não revelam a violência sofrida.

## Introduction

Global data collected in a systematic review showed that 35.6% of women aged 15 years and older reported having experienced physical and/or sexual partner violence, or sexual violence by a non-partner; this led the World Health Organization (WHO) to declare that “violence against women is a public health problem of epidemic proportions”. 1 The WHO highlighted that if the same measures of sexual violence (SV) were measured together, during childhood and adulthood, in all its forms and by all perpetrators (partners and non-partners), the prevalence rates would be much higher. 1 Other publications have shown a high prevalence of SV against girls and female adolescents. 2 3 In Brazil, 179,278 victims of sexual violence aged 0 to 19 years were reported in the period from 2017 to 2020, most of them females. 4

A couple of outcomes after SV against women are unwanted pregnancy (UP) and sexually transmitted infections (STIs). 1 2 3 A prospective study with adolescents aged 13 to 17 years described that, after 4 to 5 months of sexual aggression, 4/105 (4%) adolescents had become pregnant, 14/119 (12%) had a STI, and 9/107 (8%) reported new sexual assault. 5 Unwanted pregnancy leads to increased rates of unsafe abortion and its sequelae, and STIs can limit the women's reproductive future. 1 2 3 Particularly, when abuse occurs at a very young age, long-term complications such as physical and mental disorders and the development of high-risk behaviors are described. 5 6 7 In the first weeks after abuse, physical pain, and mental disorders—such as posttraumatic stress disorder, depressive and anxious symptoms—are described in most women. 8 9 Studies describe forms of self-harm, such as cutting, and suicidal ideation/behavior. 1 2 3 9 A study described, in girls aged 12 to 17 years, an association between sexual abuse suffered in childhood and the development of the use of substances, alcohol and other drugs, and aggressive behavior; and supporting the need to address the issue of risk behaviors during the follow-up of survivors of sexual abuse. 7 These situations can decrease women's quality of life. Women survivors of SV describe, in the long term, higher sexual dysfunction scores and lower scores in quality of life. 10

In order to reduce immediate complications, prophylactic measures have been established in emergency care services, which should be offered within the first 72 hours after SV, by administering emergency contraception, a single dose of antibiotics and postexposure prophylaxis (PEP) against human immunodeficiency virus (HIV) for 28 days. 11 12 Health services should provide protocols for short and long-term mental health support to survivors of SV, in order to avoid or reduce late complications. 12 Although this care contributes to lessening SV-related harm, studies have described low adherence rates both for treatment in the 1st month and for subsequent outpatient follow-up of up to 6 months. 13 14 15 16 17 18

Few studies have specifically evaluated the adherence of surviving adolescents to these protocols. 15 16 19 A study evaluated female survivors of SV aged 12 to 19 years and described that 44/131 (33.6%) completed treatment within the 1st month. 19 The main factors associated with adherence were the victim being white and the aggressor being unknown. 19

The aim of the present study was to evaluate the loss to follow-up and associated variables among adolescent female survivors of SV who received emergency care in the period from 2011 to 2018 at a university reference service. The analysis was performed after the emergency care and during outpatient follow-up before the standard time predicted of 6 months.

## Methods

This was a retrospective cohort study conducted at the Women's Health Care Center, School of Medical Sciences, University of Campinas (UNICAMP). The study was approved by the institutional research ethics committee (REC) under CAAE 20479819.4.0000.5404. Due to the design of this study, we asked the REC to waive the application of the consent form for this research, which was accepted. We followed all items in the strengthening of the communication of observational studies in epidemiology (STROBE).

Our university hospital is a regional reference for the care of female survivors of SV in the city of Campinas, SP, Brazil, which provides services to a coverage area for approximately 4.0 million inhabitants. Since the late 1990s, care for survivors of SV and its injuries has been standardized and must be dispensed by the Unified Health System (SUS, in the Portuguese acronym). 20 21 Our service takes care of female survivors in the first 6 months after SV, providing emergency care and outpatient 6-month follow-up with a multidisciplinary team, as provided for in the Technical Standard. 21

All women seeking the service in the first 72 hours after SV receive the prophylaxis for bacterial and viral STI, as well as emergency contraceptive; those that arrive between 72 hours and 5 days receive emergency contraception. After emergency care, outpatient consultations are scheduled to follow 6 months with a multidisciplinary team. The loss to follow-up after emergency care was defined when adolescents performed emergency care after SV and never returned to the service. The loss of outpatient follow-up was defined for adolescents who underwent emergency care after SV and returned for outpatient follow-up but did not complete 6 months of multidisciplinary care.

This study was conducted exclusively with data from the review of electronic and physical medical records of female survivors of SV aged 10 to 18 years, who underwent emergency care from January 1 ^st^ , 2011, to December 31 ^st^ , 2018. We collected data from emergency care and outpatient follow-up performed by the multidisciplinary team during the 6 months. The last adolescent included had her medical record reviewed until July 1 ^st^ , 2019, when data collection was completed.

The variables were sociodemographic (age group [10–14 years old; 15–18 years old]), self-reported ethnicity (white/non-white); education (≤ 8 years/> 8 years); intellectual disability (ID) (yes/no); marital status (single/cohabitating with partner), occupation (student/employee/without occupation), and religion (Pentecostal-Evangelical tradition/Catholic/no religion/other religion)]; personal background (history of initiated sexual life (yes/no); previous SV; history of mental disorders [psychiatric disorder/other disorder/suicidal behavior/no disorder]); characteristics of the SV (acute abuse [an event that is not repeated] or chronic abuse [when the aggression is repeated over time and is perpetrated by the same aggressor/aggressors]; aggressor (known/unknown); number of aggressors (single/multiple); type of violence suffered (vaginal/oral/anal); suffered some intimidation (yes/no); *blackout* during the aggression (yes/no); psychoactive substance (PAS) consumption before the event (alcohol; other PAS); SV perception (yes/no/she does not know/she lied about the violence); disclosure about SV (yes/no); received support from someone (yes/no) and time passed until emergency care in hours.

To assess the loss of outpatient follow-up before 6 months, we used the previous variables and those that measured the *reactions triggered after abuse* : physical disorders (sleep disturbs/appetite disturbs/others disturbs [physical disposition, gastrointestinal and urinary symptoms]); mental disorders (anxious symptoms/depressive symptoms/suicidal behavior/suicide attempt/flashbacks); social reactions (social avoidance/changes in daily routine [irregular bedtime/wake up and meal times/missed school/work/other activities; not being home alone at home/not going go out alone]/change of address/change of city/change of school); and psychotropic prescription needs during follow-up.

To compare categorical variables between lost to follow-up and non-lost to follow-up groups, we used the Chi-square or Fisher exact test. We used univariate and multiple logistic regression analysis with stepwise selection criteria to determine the variables associated with loss to follow-up at two times: after emergency care and before the end of the 6-month outpatient follow-up. We calculated the odds ratio (OR) for loss to follow-up with a 95% confidence interval (CI). The significance level was 5%. We used the Statistical Analysis System for Windows, version 9.2 (SAS Institute Inc, 2002–2008, Cary, NC, USA).

## Results

**Fig. 1:**
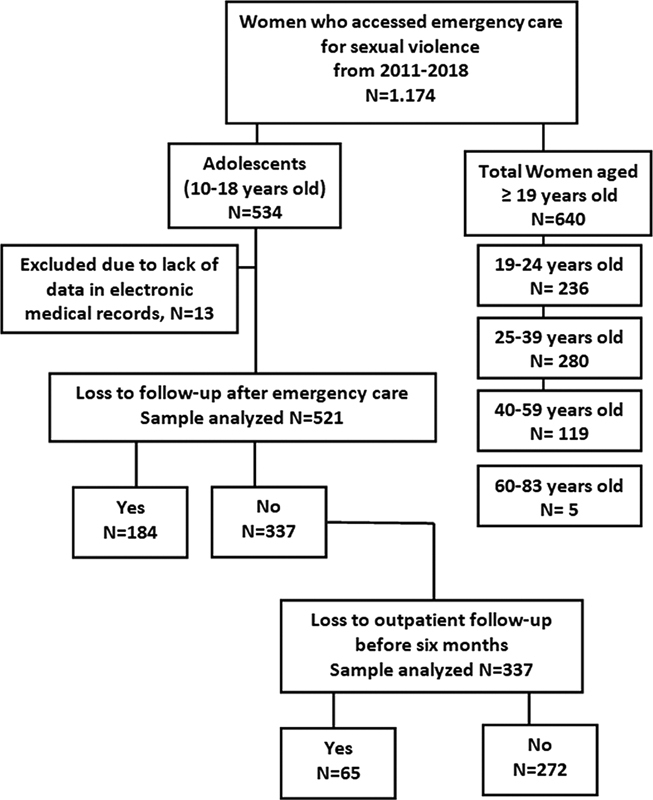
Flowchart of emergency care digitized from the hospital database according to the age characteristics of the women treated between 2011 and 2018.

During the period from January 1 ^st^ , 2011, to December 31 ^st^ , 2018, 1,174 women survivors of SV received emergency care at our service, 534 (45.5%) of whom were adolescents aged 10 to 18 years. Thirteen (2.4%) adolescents were excluded due to a lack of data recorded in the medical records ( Figure 1 ).

Table 1 shows some sociodemographic characteristics of the 521 adolescents who made up this sample. More than half of the adolescents (53.5%) were aged between 15 to 18 years, 64.2% self-reported white ethnicity, and were equally distributed between ≤ 8 and > 8 years of formal education. Most of the adolescents (92.1%) were single, 85.3% studying, and 4.6% had ID. Less than 10% of them lived with their partner, were employed; or had no occupation, that is, they were not studying or working at the time they suffered SV ( Table 1 ). Of the 521 adolescents, 249 (47.7%) were lost to follow-up at some point during the follow-up; 184/521 (35.3%) adolescents were lost to follow-up after emergency care, and 65/521 (12.4%) adolescents who started the outpatient follow-up were lost to follow-up before completing 6 months ( Table 1 ).

**Table 1:** Sociodemographic characteristics of adolescent victims of sexual violence who received emergency care in the period from 2011 to 2018 and frequency of loss to follow-up soon after emergency care and outpatient follow-up loss < 6 months

Sociodemographic characteristics	n	%
Age group (n = 521)		
10–14 years old	242	46.4
15-–18 years old	279	53.5
Self-reported ethnicity (n = 517)		
White	332	64.2
Non-White	185	35.8
Education (n = 517)		
≤ 8 years	260	50.3
> 8 years	257	49.7
Intellectual disability (n = 521)		
Sim	24	4.6
Nao	497	95.4
Marital status (n = 521)		
Single	480	92.1
Cohabiting with a partner	41	7.8
Occupation (n = 491)		
Student	419	85.3
Employee	31	6.3
No occupation	41	8.3
Religion		
Pentecostal-Evangelical tradition	199	41.4
Catholic	149	31.0
No religion	104	21.6
Other religion	28	5.8
Loss to follow-up (n = 521)		
After emergency care	184	35.3
Before completing 6 months	65	12.4
Completed follow-up	272	52.2

Table 2 shows the results of the regression analysis for abandonment of follow-up after emergency care. The univariate regression analysis showed that cohabiting with a partner, not having a religion or having a Catholic religion, not having experienced a blackout during the assault, not having initiated sexual life before the SV, having no history of previous SV, not having disclosed the violence to anyone, and not having received support from someone were characteristics associated with loss to follow-up after receiving emergency care ( Table 2 ). After multiple logistic regression, adolescents who lived with a partner remained associated with a higher risk of loss to follow-up after emergency care, with OR 5.94 (95% CI; 2.49–14.20) in relation to those who did not. The risk of loss to follow-up after emergency care was also higher for adolescents who reported not having a religion (OR 2.38 [95% CI; 1.29–4.38]) and having a Catholic religion (OR 2.11 [95% CI; 1.17–3.78]) in relation to those who had a Protestant religion; and those who reported not having disclosed the aggression to anyone had twice the risk (OR 2.07 [95% CI; 1.25–3.44]) in relation to those who had told ( Table 2 ). No other variables were associated with loss to follow-up after emergency care (data not shown).

**Table 2:** Comparison of adolescents who lost or not follow-up after emergency care and variables associated with discontinuing according to sociodemographic characteristics, personal history, type of violence suffered, attitudes after the event and time until emergency care

Variables	Loss to follow-up after emergency care						
	Comparative analysis			Univariate analysis c		Multivariate logistic regression analysis d	
	Yes	No	P	P c	OR (IC 95%)	P d	OR (IC 95%)
Age group (n = 521)			0.315 a				
10–14 years old	80 (43.4)	162 (48.0)			Ref.		
15–18 years old	104 (56.5)	175 (51.9)		0.315	1.20 (0.84–1.73)	NS	
Self-reported ethnicity (n = 517)			0.634 a				
White	120 (65.5)	212 (63.4)			Ref.		
Non-white	63 (34.4)	122 (36.5)		0.634	0.91 (0.63–1.33)	NS	
Education (n = 517)			0.996 a				
≤ 8 years	92 (50.2)	168 (50.3)			Ref.		
> 8 years	91 (49.7)	166 (49.7)		0.996	1.00 (0.70–1.44)	NS	
Intellectual disability (n = 521)			0.505 a				
No	174 (94.5)	323 (95.8)			Ref.		
Yes	10 (5.4)	14 (4.1)		0.506	1.33 (0.58–3.05)	NS	
Marital status (n = 521)			< 0.001 a				
Single	155 (84.2)	325 (96.4)			Ref.		Ref.
Cohabiting with a partner	29 (15.7)	12 (3.5)		< 0.001	5.07 (2.52–10.20)	< 0.001	5.94 (2.49–14.20)
Occupation (n = 491)			0.347 a			NS	
Student	142 (84.5)	277 (85.7)			Ref.		
Employee	14 (8.3)	17 (5.2)		0.207	1.61 (0.77–3.35)	NS	
No occupation	12 (7.1)	29 (8.9)		0.550	0.81 (0.40–1.63)	NS	
Religion (n = 480)			< 0.001 a				
Protestant	52 (30.0)	147 (47.9)			Ref.		Ref.
Catholic	68 (39.3)	81 (26.4)		< 0.001	2.37 (1.51–3.73)	0.013	2.11 (1.17–3.78)
No religion	44 (25.4)	60 (19.5)		0.004	2.07 (1.26–3.42)	0.005	2.38 (1.29–4.38)
Other religion	9 (5.2)	19 (6.2)		0.503	1.34 (0.57–3.15)	0.558	0.67 (0.18–2.53)
Sexual activity initiated (n = 506)			0.034 a				
Yes	43 (24.1)	109 (33.2)			Ref.		
No	135 (75.8)	219 (66.7)		0.034	1.56 (1.03–2.36)	NS	
History of mental disorder (n = 428)			0.249 a				
No	100 (90.1)	272 (85.8)			Ref.		
Yes	11 (9.9)	45 (14.2)		0.252	0.67 (0.33–1.34)	NS	
Previous mental disorder (n = 56)			0.308 b				
No disorder			^−^		Ref.		
Psychiatric disorder	5 (45.4)	24 (53.3)		0.261	0.57 (0.21–1.53)	NS	
Suicidal behavior	2 (18.1)	10 (22.2)		0.437	0.54 (0.12–2.53)	NS	
Other disorder	4 (36.3)	11 (24.4)		0.985	0.99 (0.31–3.18)	NS	
History of sexual violence (n = 511)			0.033 a				
Yes	22 (12.2)	65 (19.6)			Ref.		
No	158 (87.8)	266 (80.3)		0.035	1.75 (1.04–2.96)	NS	
Abuse (n = 521)			0.240 a				
Single abuse	177 (96.2)	316 (93.7)			Ref.		
Chronic abuse	7 (3.8)	21 (6.2)		0.245	0.60 (0.25–1.43)	NS	
Known aggressor (n = 520)			0.687 a				
Yes	96 (52.4)	183 (54.3)			Ref.		
No	87 (47.5)	154 (45.7)		0.687	1.08 (0.75–1.55)	NS	
Number of aggressors (n = 520)			0.215 a				
Single	162 (88.5)	285 (84.5)			Ref.		
Multiple	21 (11.4)	52 (15.4)		0.161	0.67 (0.38–1.17)	NS	
Some intimidation (n = 491)			0.381 a				
No	17 (10.4)	32 (9.7)			Ref.		
Yes	135 (82.8)	283 (86.2)		0.735	0.90 (0.48–1.67)	NS	
Does not know	11 (6.7)	13 (3.9)		0.359	1.59 (0.59–4.31)	NS	
Vaginal aggression (n = 521)			0.985 a				
No	67 (36.4)	123 (36.5)			Ref.		
Yes	117 (63.6)	214 (63.5)		0.985	1.00 (0.69–1.46)	NS	
Oral aggression (n = 521)			0.154 a				
No	149 (80.9)	289 (85.7)			Ref.		
Yes	35 (19.0)	48 (14.2)		0.156	1.41 (0.88–2.28)	NS	
Anal aggression (n = 521)			0.209 a				
No	162 (88.0)	283 (83.9)			Ref.		
Yes	22 (11.9)	54 (16.0)		0.210	0.71 (0.42–1.21)	NS	
Alcohol use before aggression (n = 469)			0.277 a				
No	149 (87.6)	251 (83.9)			Ref.		
Yes	21 (12.3)	48 (16.0)		0.055	0.56 (0.31–1.01)	NS	
PAS use before aggression (n = 420)			0.236 a				
No	152 (97.4)	251 (95.0)			Ref.		
Yes	4 (2.5)	13 (4.9)		0.233	0.56 (0.22–1.45)	NS	
*Blackout* during the aggression (n = 515)			0.009 a				
Yes	28 (15.4)	85 (25.4)			Ref.		
No	153 (84.5)	249 (74.5)		0.029	1.70 (1.05–2.74)	NS	
Sexual violence perception (n = 521)		0.061 b					
Yes	172 (93.5)	293 (86.9)			Ref.		
No	5 (2.7)	28 (8.3)		0.016	0.30 (0.12–0.80)	NS	
She does not know	4 (2.1)	7 (2.1)		0.966	0.97 (0.28–3.37)	NS	
She lied about the violence	3 (1.6)	9 (2.6)		0.401	0.57 (0.15–2.13)	NS	
Disclosure about sexual violence (n = 414)		< 0.001 a					
Yes	58 (50.8)	222 (74.0)			Ref.		Ref.
No	56 (49.1)	78 (26.0)		< 0.001	2.75 (1.75–4.30)	0.005	2.07 (1.25–3.44)
Received support from someone (n = 361)			0.023 a				
Yes	48 (64.0)	220 (76.9)			RRef.		
No	27 (36.0)	66 (23.0)		0.023	1.88 (1.09–3.24)	NS	
Time until emergency care			0.871 a				
≤ 72 hours	123 (69.1)	237 (70.5)			Ref.		
> 72h––5 days	15 (8.4)	27 (8.0)		0.842	1.07 (0.55–2.09)	NS	
> 5 days–6 months	40 (22.4)	72 (21.4)		0.764	1.07 (0.69–1.67)	NS	

Abbreviations: CI, confidence interval; NS, not significant; OR, odds ratio; Ref, reference level.

a Chi-squared test.

b Fisher exact test.

c Univariate logistic regression analysis ( *n = 521* ; loss to follow-up after emergency care no, *n = 337* ; loss to follow-up after emergency care yes, *n = 184* ).

d Multivariate logistic regression analysis with stepwise variable selection criteria ( *n = 337* ; loss to follow-up after emergency care no, *n = 271* ; loss to follow-up after emergency care yes, *n = 107)* .

The variables associated with abandonment of outpatient follow-up before 6 months after regression analysis are shown in Table 3 . The univariate analysis showed that the variables that increased the chances of loss to follow-up were lying about the SV suffered, not having disclosed the aggression to anyone, not having received support from someone, not having presented the need to prescribe psychotropic drugs, and not having presented reactions of physical disorders, sleep disorders, mental disorders, anxiety symptoms, social changes and social avoidance behaviors triggered after the violence ( Table 3 ). After multiple logistic regression analysis, only two variables remained associated with loss to outpatient follow-up before 6 months: adolescents who did not have mental disorders and did not report social changes were more likely to discontinue this follow-up before 6 months, (OR 2.72 [95% CI; 1.36–5.46]) and (OR 2.33 [95% CI; 1.09–4.99]), respectively, in relation to the adolescents who presented these reactions ( Table 3 ). No other variables were associated with loss to follow-up before the end of the 6 months (data not shown).

**Table 3:** Comparison between adolescents who were lost and those who were not to outpatient follow-up before 6 months and variables associated with dicontinuity according to sociodemographic characteristics, personal history, type of violence suffered, attitudes after the event, time until emergency care, and physical and mental disorders as well as social reactions triggered after abuse

Variables	Loss to follow-up before 6 months						
	Comparative analysis			Univariate analysis c		Multivariate logistic regression analysis d	
	Yes	No	P	P c	OR (CI 95%)	P d	OR (CI 95%)
Age (n = 337)			0.447 a				
10–14 years old	34 (52.3)	128 (47.0)			Ref.		
15–18 years old	31 (47.7)	144 (52.9)		0.447	0.81 (0.47–1.39)	NS	
Self-reported ethnicity (334)			0.857 a				
White	40 (62.5)	172 (63.7)			Ref.		
Non–white	24 (37.5)	98 (36.3)		0.857	1.05 (0.60–1.85)	NS	
Education (n = 334)			0.290 a				
≤ 8 years	36 (56.2)	132 (48.9)			Ref.		
> 8 years	28 (43.7)	138 (51.1)		0.291	0.74 (0.43–1.29)	NS	
Intellectual disability (n = 337)			0.485 b				
No	61 (93.8)	262 (96.3)			Ref.		
Yes	4 (6.1)	10 (3.6)		0.374	1.72 (0.52–5.66)	NS	
Marital status (n = 337)			0.474 b				
Single	64 (98.4)	261 (95.9)			Ref.		
Cohabiting with a partner	1 (1.5)	11 (4.0)		0.346	0.37 (0.05–2.92)	NS	
Occupation (323)			0.826 a				
Student	51 (87.9)	226 (85.2)			Ref.		
Employee	3 (5.1)	14 (5.2)		0.540	0.71 (0.24–2.13)	NS	
No occupation	4 (6.9)	25 (9.4)		0.937	0.95 (0.26–3.43)	NS	
Religion (307)			0.303 a				
Catholic	19 (31.6)	62 (25.1)			Ref.		
Pentecostal-Evangelical tradition	30 (50.0)	117 (47.3)		0.592	0.84 (0.44–1.61)	NS	
No religion	10 (16.6)	50 (20.2)		0.326	0.65 (0.28–1.53)	NS	
Other religion	1 (1.6)	18 (7.3)		0.107	0.18 (0.02–1.45)	NS	
Sexual activity initiated (n = 328)			0.227 a				
No	38 (60.3)	181 (68.3)			Ref.		
Yes	25 (39.6)	84 (31.7)		0.228	1.42 (0.80–2.50)	NS	
History of mental disorder (317)			0.106 a				
No	51 (92.7)	221 (84.3)			Ref.		
Yes	4 (7.2)	41 (15.6)		0.115	0.42 (0.15–1.23)	NS	
Previous mental disorder (317)			0.506 b				
None					Ref.		
Psychiatric disorder	2 (50.0)	22 (53.6)		0.802	0.87 (0.28–2.65)	NS	
Suicidal behavior	–	10 (24.4)		0.131	0.20 (0.01–3.55)	NS	
Other disorder	2 (50.0)	9 (21.9)		0.113	0.19 (0.01–3.23)	NS	
History of sexual violence (n = 331)			0.842 a				
No	52 (81.2)	214 (80.1)			Ref.		
Yes	12 (18.7)	53 (19.8)		0.842	0.93 (0.47–1.87)	NS	
Abuse (337)			0.572 b				
Single abuse	60 (92.3)	256 (94.1)			Ref.		
Chronic abuse	5 (7.7)	16 (5.8)		0.589	1.33 (0.47–3.78)	NS	
Known aggressor (337)			0.846 a				
Yes	36 (55.3)	147 (54.0)			Ref.		
No	29 (44.6)	125 (45.9)		0.846	0.95 (0.55–1.63)	NS	
Number of aggressors (337)			0.711 a				
Single	54 (83.0)	231 (84.9)			Ref.		
Multiple	11 (16.9)	41 (15.0)		0.615	1.21 (0.58–2.51)	NS	
Some intimidation			0.652 a				
No	8 (12.7)	24 (9.0)			Ref.		
Yes	53 (84.1)	230 (86.8)		0.397	0.69 (0.29–1.62)	NS	
Does not know	2 (3.1)	11 (4.1)		0.486	0.55 (0.10–3.00)	NS	
Vaginal aggression (337)			0.621 a				
No	22 (33.8)	101 (37.1)			Ref.		
Yes	43 (66.1)	171 (62.8)		0.621	1.15 (0.65–2.04)	NS	
Oral aggression (337)			0.198 a				
No	59 (90.7)	230 (84.5)			Ref.		
Yes	6 (9.2)	42 (15.4)		0.203	0.56 (0.23–1.37)	NS	
Anal aggression (337)			0.551 a				
No	53 (81.5)	230 (84.5)			Ref.		
Yes	12 (18.4)	42 (15.4)		0.551	1.24 (0.61–2.52)	NS	
Alcohol use before aggression (299)			0.270 a				
No	41 (78.8)	210 (85.0)			Ref.		
Yes	11 (21.1)	37 (14.9)		0.671	0.84 (0.39–1.85)	NS	
PAS use before aggression (264)			0.460 b				
No	41 (93.1)	210 (95.4)			Ref.		
Yes	3. (6.8)	10 (4.5)		0.418	1.56 (0.53–4.54)	NS	
*Blackout* about the aggression (n = 334)			0.387 a				
No	45 (70.3)	204 (75.5)			Ref.		
Yes	19 (29.7)	66 (24.4)		0.927	1.03 (0.55–1.92)	NS	
Sexual violence perception (n = 337)			0.004 b				
Yes	50 (76.9)	243 (89.3)			Ref		
No	7 (10.7)	21 (7.7)		0.298	1.62 (0.65–4.02)	NS	
She does not know	2 (3.0)	5 (1.8)		0.435	1.94 (0.37–10.30)	NS	
She lied about the violence	6 (9.23)	3 (1.1)		0.002	9.72 (2.35–40.17)	NS	
Disclosure about sexual violence (n = 300)			0.009 a				
Yes	31 (59.6)	191 (77.0)			Ref		
No	21 (40.4)	57 (23.0)		0.010	2.27 (1.21–4.25)	NS	
Received support from someone (n = 286)			0.009 a				
Yes	30 (62.5)	190 (79.8)			Ref		
No	18 (37.5)	48 (20.1)		0.011	2.38 (1.22–4.62)	NS	
Time until emergency care (n = 336)			0.764 b				
≤ 72 hours	50 (76.9)	187 (69.0)			Ref.		
> 72 hours–5 days	3 (4.6)	24 (8.8)		0.230	0.47 (0.14–1.62)	NS	
> 5 days–6 months	12 (18.4)	60 (22.1)		0.412	0.75 (0.37–1.50)	NS	
Reactions triggered after abuse (n = 302)							
Some physical disorder (n = 302)			0.011 a				
Yes	17 (32.1)	128 (51.4)			Ref		
No	36 (67.9)	121 (48.6)		0.012	2.24 (1.20–4.20)	NS	
Sleep disorders (n = 302)			0.043 a				
Yes	15 (28.3)	108 (43.3)		–	Ref		
No	38 (71.7)	141 (56.6)		0.045	1.94 (1.02–3.71)	NS	
Appetite disorder (n = 302)			0.117 a				
No	46 (86.8)	192 (77.1)			Ref.		
Yes	7 (13.2)	57 (22.9)		0.123	0.51 (0.22–1.20)	NS	
Other physical disorder (n = 302)			0.544 b				
No	51 (96.2)	232 (93.1)			Ref.		
Yes	2 (3.7)	17 (6.8)		0.291	0.45 (0.10–1.98)	NS	
Some mental disorder (n = 302)			< 0.001 a				
Yes	22 (41.5)	174 (69.8)			Ref		Ref
No	31 (58.5)	75 (30.1)		< 0.001	3.27 (1.78–6.02)	0.005	2.72 (1.36–5.46)
Anxiety symptoms (n = 302)			0.013 a				
Yes	15 (28.3)	117 (47.0)			Ref		
No	38 (71.7)	132 (53.0)		0.014	2.25 (1.18–4.29)	NS	
Depressive symptoms			0.140 a				
No	48 (90.5)	205 (82.3)			Ref.		
Yes	5 (9.4)	44 (17.6)		0.147	0.49 (0.18–1.29)	NS	
Suicide attempt			0.415 b				
No	50 (94.3)	241 (96.8)			Ref.		
Yes	3 (5.6)	8 (3.2)		0.394	1.81 (0.46–7.05)	NS	
Suicidal behavior (n = 302)			0.589 b				
No	50 (94.3)	227 (91.1)			Ref.		
Yes	3 (5.6)	22 (8.8)		0.450	0.62 (0.18–2.15)	NS	
Flashbacks (n = 302)			0.047 a				
No	48 (90.5)	196 (78.7)			Ref.		
Yes	5 (9.4)	53 (21.3)		0.054	0.39 (0.15–1.02)	NS	
Some social change (n = 302)			< 0.001 a				
Yes	33 (62.2)	213 (85.5)			Ref		Ref
No	20 (37.7)	36 (14.4)		< 0.001	3.59 (1.86-6.93)	0.029	2.33 (1.09–4.99)
Social avoidance (n = 302)			0.009 a				
Yes	7 (13.2)	77 (30.9)		–	Ref		
No	46 (86.8)	172 (69.1)		0.006	2.79 (1.34–5.81)	NS	
Changes in daily routine (n = 302)			0.477 a				
No	45 (84.9)	201 (80.7)			Ref.		
Yes	8 (15.1)	48 (19.2)		0.478	0.75 (0.33–1.68)	NS	
Changes of address (n = 302)			1.000 b				
No	49 (92.4)	227 (91.1)			Ref.		
Yes	4 (7.5)	22 (8.8)		0.762	0.84 (0.28–2.55)	NS	
Changes of school (n = 302)			0.719 b				
No	50 (94.3)	238 (95.5)			Ref.		
Yes	3 (5.6)	11 (4.4)		0.697	1.30 (0.35–4.82)	NS	
Changes of city or state (n = 302)			0.540 b				
No	52 (98.1)	246 (98.8)			Ref.		
Yes	1 (1.9)	3 (1.20)		0.695	1.58 (0.16–15.47)	NS	
Psychotropic prescription needs (n = 282)			0.007 a				
Yes	9 (18.7)	92 (39.3)			Ref		
No	39 (81.2)	142 (60.7)		0.009	2.81 (1.30–6.07)	NS	

Abbreviations: CI, confidence interval; NS, not significant; OR, odds ratio; ref, reference level.

a Chi-square test.

b Fisher exact test.

c Univariate logistic regression analysis ( *n = 337;* loss to follow-up before 6 months no, *n = 272* ; loss to follow-up before 6 months yes, *n = 65* ).

d Multivariate logistic regression analysis with stepwise variable selection criteria ( *n = 277* ; loss of outpatient follow-up no, *n = 230* ; loss of outpatient follow-up yes, *n = 47* ).

## Discussion

Almost half of the adolescents, 249/521 (47.7%), who received emergency care did not complete the proposed outpatient follow-up. Of 521 adolescents, 184 (35.3%) discontinued after emergency care and did not start outpatient follow-up; another 65 (12.4%) adolescents were lost to outpatient follow-up before 6 months. We consider these loss-to-follow-up rates to be high, especially for the age group studied.

Other studies of survivors of SV have shown higher rates of discontinuation before the age of 6 months. 13 14 15 16 17 18 A prospective study carried out from 1988 to 1990 in a university service in Vancouver, Canada, with women aged over 16 years treated in emergency care after SV reported that only 61/296 (21%) could be traced to 6 months. 13 A retrospective study analyzed data from 1,695 adult victims of sexual abuse treated in emergency care in Barcelona during the period from 2006 to 2015 and reported that, among the 883 who received prophylactic treatment for HIV, only 284 (32%) completed the 6-month follow-up. 17 One study conducted in Brazil evaluated survivors of SV who received emergency care from 2001 to 2013 and described that 110/199 (55.2%) discontinued outpatient follow-up before 6 months. 14 Another Brazilian retrospective study, conducted between 2007 and 2016, that aimed to assess the care provided to female survivors of SV aged 11 to 77 years reported that 167/444 (37.6%) women did not return within the 1st month, and 284/444 (64%) discontinued before completing the follow-up of 90 days. 18

The lower loss to follow-up found in our study compared to those mentioned was probably due to differences between the samples. While our study included only adolescent females up to 18 years of age, the others included populations of survivors of different ages. It is important to highlight that the Brazilian legislation determines that the legal guardian of minors under 19 years of age be held accountable when the rights linked to public policies are not fulfilled, including the treatment and monitoring of victims of SV. 21 Therefore, by virtue of the law, the adolescent population usually complies with the care provided more frequently, at the risk of legal measures that may be proposed by the Guardianship Council and executed by the Judge of Childhood and Adolescence. 21 In addition, our service routinely makes phone calls to teenagers who have missed appointments to reschedule them before reporting the absence to the Guardianship Council, which also increases the frequency of appointments. We found a single prospective study of sexually assaulted female adolescents who received emergency care at 18 hospital centers in Ontario, Canada, and that assessed adherence to PEP treatment. 22 The results showed that 131/307 (42.7%) adolescents agreed to use the treatment, and only 44/131 (33.6%) completed the planned 28-day treatment. 22 Other clinic-based studies in Zimbabwe and Kenya with victims seen in the emergency room, and which included the description of frequency of adolescents and females, described even lower adherence rates, of 8% and 1.4%, respectively, for follow-up periods of up to 60 days. 15 16

Our results show that the loss to follow-up was higher right after emergency care. We do not have information regarding whether the adolescents who did not return completed the prophylaxis or if they became pregnant. However, as our service is the regional reference and no adolescent returned pregnant, we believe that this complication did not occur in the adolescents in this sample. The multivariate regression analysis showed that the chance of not starting outpatient follow-up was six times increased in adolescents who lived with a partner and twice as high in cases of non-disclosure. Although intimate partner violence is described as a contributing factor to discontinuation of care, 16 in our results, having a known aggressor was not associated with loss to follow-up at any of the studied time points.

However, the opinion of the partner and/or family members may have influenced adherence to care, as described by other authors, including possible added fear due to the proximity of living with the aggressor. 22 Studies carried out in Vancouver, Canada (1992), and Mombasa (2019) and Nairobi (2022), in Kenya, showed that both family interference and proximity to the aggressor can influence adherence to follow-up. 13 16 22 The study carried out in Nairobi with 28 multidisciplinary health professionals, experienced in providing care to children and adolescents who have survived SV, sought to assess the difficulties and challenges of providing quality care. 22 Professionals described that family interference was a barrier for adolescents to disclose SV and to have access to health care and justice; in addition, family interference influenced the quality of care and loss to follow-up. 22

In patriarchal cultures such as Brazil, women are socially stigmatized after suffering SV, which certainly favors non-disclosure on the part of survivors about the violence. Moreover, they do not want to remember the event, reducing the chance that they will attend the outpatient follow-up. Adolescents who reported not having a religion and having a Catholic religion were twice as likely to be lost to follow-up compared to those who had a Protestant religion. Although the majority of the Brazilian population calls itself Catholic, it is known that this affiliation is associated with considerable flexibility in religious practice and in the feeling of belonging, which comes close to having no religion. 22 23 On the contrary, in Protestant denominations, in general, members attend services more frequently and share values in the family nucleus. 23 24 Thus, it is possible that the association between the variables not having a religion/being Catholic and non-adherence to outpatient follow-up may be due to a less effective support network.

The loss of outpatient follow-up was less frequent and considered by the authors to have less impact on treatment, since only two variables were shown to be associated with loss of outpatient follow-up: not having a mental illness and not reporting social reactions. We conclude that the adolescents who discontinued the outpatient follow-up before 6 months were those who no longer needed mental health support and those who were not forced to change their life routine, that is, they did not feel threatened, frightened, or rejected in the social environment. On the other hand, we consider it possible that the more intense situations of suffering favored adherence and the need to persist in the follow-up, while the need to help to change schools, housing or to achieve protective actions may have contributed to increasing the bond with the team and adherence to the follow-up until the 6th month. These results agree with a study describing that survivors who received at least one psychological consultation were more likely to finish treatment in the first month and to remain in follow-up for 6 months. 14 It also agreed with a study carried out only with female adolescents, which described that being moderately or very anxious, the attitude of encouragement given by the provider and being a student were factors associated with acceptance of care. 22

At the time of SV, just under 10% of the adolescents lived with their partner, were employed; or had no occupation, which means that they neither studied nor worked. These characteristics increase the vulnerability of the individual to suffer SV. An early age for both marriage and paid employment can decrease the conditions for better educational preparation and to develop social skills, and, moreover, increases susceptibility to intimate partner violence 3 ; the lack of occupation can predispose to the risk of suffering SV both inside and outside the space of the household and predispose to the development of higher risk behaviors. 22 In this sample, the rate of 4.6% of adolescents with ID was higher than that described in the general population, around 1%, 25 but it was not associated with a greater loss to follow-up, as described by other authors. 13

We consider the retrospective nature of this study to be its main limitation. Certainly, we have a lot of missing information not collected in during appointments and added to the retrospective data analysis, which may have induced some bias in the results. During the consultations, we did not ask in detail about religion and its practice, nor have we evaluated the possible interference of family members/close people in the adolescents' attitudes and in their health care. Another limitation of this study was the lack of data on family income and social status of the adolescents followed up at our service. On the other hand, to our knowledge, this is the 1st study to assess the abandonment of follow-up in adolescent females during a 6-month follow-up period.

## Conclusion

Prospective studies should be carried out to better understand the individual factors that may lead to loss to follow-up in adolescent female survivors of SV. Particularly, adolescents who live with their partner and those who report not having anyone to whom to disclose the abuse should receive specific care in the emergency room to increase adherence to follow-up.

## References

[1] World Health Organization Global and regional estimates of violence against women: prevalence and health effects of intimate partner violence and nonpartner sexual violence. [Internet] Geneva World Health Organization 2013[cited 2022 Nov 30]. Available from:https://www.who.int/publications/i/item/9789241564625

[2] United Nations International Children's Emergency Fund (UNICEF) Hidden in Plain Sight: A statistical analysis of violence against children. [Internet] Geneva World Health Organization 2014[cited 2022 Nov 30]. Available from:https://www.unicef.org/documents/hidden-plain-sight-statistical-analysis-violence-against-children

[3] Lehtimaki S Schwalbe N Adolescent health: the missing population in universal health coverage [Internet] Geneva World Health Organization, UNICEF, Plan International, International Association for Adolescent Health (IAAH), The Partnership for Maternal, Newborn & Child Health, and Child Health Initiative 2018[cited 2022 Nov 30]. Available from:https://www.unicef.org/media/58171/file

[4] United Nations Children's Fund (UNICEF), Brazilian Public Security Forum Overview of lethal and sexual violence against children and adolescents in Brazil Brasília UNICEF 2021[cited 2022 Nov 30]. Available from:https://www.unicef.org/brazil/media/17341/file/panorama-lethal-sexual-violence-against-children-adolescents-in-brazil.pdf

[5] Khadr S Clarke V Wellings K Villalta L Goddard A Welch J Mental and sexual health outcomes following sexual assault in adolescents: a prospective cohort study Lancet Child Adolesc Health 2018 2 09 654 665 10.1016/S2352-4642(18)30202-5 30119759

[6] Howard D E Wang M Q Psychosocial correlates of U.S. adolescents who report a history of forced sexual intercourse J Adolesc Health 2005 36 05 372 379 10.1016/j.jadohealth.2004.07.007 15837340

[7] Moreland A D Walsh K Hartley C Hanson R Danielson C K Saunders B Kilpatrick D G Investigating longitudinal associations between sexual assault, substance use, and delinquency among female adolescents: results from a nationally representative sample J Adolesc Health 2018 63 03 320 326 10.1016/j.jadohealth.2018.04.002 30029849 PMC6512326

[8] Short N A Lechner M McLean B S Tungate A S Black J Buchanan J A Health care utilization by women sexual assault survivors after emergency care: Results of a multisite prospective study Depress Anxiety 2021 38 01 67 78 10.1002/da.23102 33032388 PMC7785610

[9] Torres A SB Teixeira A L Côrtes M TF Alves A C Alabarse O Azevedo R CS Fernandes A Sexual violence suffered by women in early and late Adolescence: Care provided and follow-up Rev Bras Ginecol Obstet 2022 44 07 667 677 10.1055/s-0042-1743094 35276748 PMC10032055

[10] Carreiro A V Micelli L P Sousa M H Bahamondes L Fernandes A Sexual dysfunction risk and quality of life among women with a history of sexual abuse Int J Gynaecol Obstet 2016 134 03 260 263 10.1016/j.ijgo.2016.01.024 27350228

[11] Martin S L Young S K Billings D L Bross C C Health care-based interventions for women who have experienced sexual violence: a review of the literature Trauma Violence Abuse 2007 8 01 3 18 10.1177/1524838006296746 17204597

[12] World Health Organization Responding to Intimate Partner Violence and Sexual Violence Against Women: WHO Clinical and Policy Guidelines Geneva World Health Organization 2013[cited 2022 Nov 30]. Available from:https://apps.who.int/iris/bitstream/handle/10665/85240/9789241548595_eng.pdf 24354041

[13] Herbert C P Grams G D Berkowitz J Sexual assault tracking study: who gets lost to follow-up? CMAJ 1992 147 08 1177 1184 1393931 PMC1336483

[14] Nisida I VV Boulos M C da Silva L MB Mayaud P Avelino-Silva V I Segurado A C Predictors of adherence to HIV post-exposure prophylaxis and retention in care after an episode of sexual violence in Brazil AIDS Patient Care STDS 2019 33 09 399 405 10.1089/apc.2019.0080 31386552

[15] Harrison R E Pearson L Vere M Chonzi P Hove B T Mabaya S Care requirements for clients who present after rape and clients who presented after consensual sex as a minor at a clinic in Harare, Zimbabwe, from 2011 to 2014 PLoS One 2017 12 09 e0184634 10.1371/journal.pone.0184634 28934344 PMC5608202

[16] Temmerman M Ogbe E Manguro G Khandwalla I Thiongo M Mandaliya K N The gender-based violence and recovery centre at Coast Provincial General Hospital, Mombasa, Kenya: An integrated care model for survivors of sexual violence PLoS Med 2019 16 08 e1002886 10.1371/journal.pmed.1002886 31374074 PMC6677296

[17] Sexual Assault Victims Study Group Inciarte A Leal L Masfarre L Gonzalez E Diaz-Brito V Lucero C Post-exposure prophylaxis for HIV infection in sexual assault victims HIV Med 2020 21 01 43 52 10.1111/hiv.12797 31603619 PMC6916272

[18] de Jesus G R Rodrigues N P Braga G C Abduch R Melli P PS Duarte G Quintana S M Assistance to victims of sexual violence in a referral service: a 10-year experience Rev Bras Ginecol Obstet 2022 44 01 47 54 10.1055/s-0041-1740474 35092959 PMC9948278

[19] Du Mont J Myhr T L Husson H Macdonald S Rachlis A Loutfy M R HIV postexposure prophylaxis use among Ontario female adolescent sexual assault victims: a prospective analysis Sex Transm Dis 2008 35 12 973 978 10.1097/OLQ.0b013e3181824f3c 18836390

[20] Brazil. Law 8.069 of July 13, 1990. Provides for the Statute of Children and Adolescents and provides other measures. Official Gazette 1990; Aug 16 Official Gazette 1990; September 27 (Rectification)Available from:http://www.biblioteca.presidencia.gov.br/publicacoes-oficiais/catalogo/collor/statute-of-the-child-and-adolescent-1990

[21] Brazil. Prevention and Treatment of Damages Resulting from Sexual Violence Against Women and Adolescents: Technical Standard Brasília Ministry of Health 2012[cited 2022 Nov 30]. Available from:https://bvsms.saude.gov.br/bvs/publicacoes/atencao_humanizada_pessoas_violencia_sexual_norma_tecnica.pdf

[22] Munala L Welle E Hohenshell E “If you report your dad, how are you going to survive”: health practitioner perspectives on quality of care for survivors of sexual violence and the challenge of family interference J Interpers Violence 2022 37(7-8):NP5294 NP5316 10.1177/088626052095962 32976039

[23] Weber S R Pargament K I The role of religion and spirituality in mental health Curr Opin Psychiatry 2014 27 05 358 363 10.1097/YCO.0000000000000080 25046080

[24] Rosmarin D H Pargament K I Koenig H G Spirituality and mental health: challenges and opportunities Lancet Psychiatry 2021 8 02 92 93 10.1016/S2215-0366(20)30048-1 32087772

[25] Ke X Liu J Intellectual disability Geneva IACAPAP 2012[2023 Apr 18]. p. 1–25. Available from:https://iacapap.org/_Resources/Persistent/59cacea32eb3387bba990aafb6f7a07f28ac6887/C.1-Intellectual-Disability.pdf

